# Handling of laundry in nursing homes in Frankfurt am Main, Germany, 2016 – laundry and professional clothing as potential pathways of bacterial transfer

**DOI:** 10.3205/dgkh000305

**Published:** 2017-11-30

**Authors:** Ursel Heudorf, Stefanie Gasteyer, Maria Müller, Nicole Serra, Tim Westphal, Claudia Reinheimer, Volkhard Kempf

**Affiliations:** 1 Public Health Department of the City of Frankfurt/Main, Germany; 2 Institute for Medical Microbiology and Infection Control, University Hospital Frankfurt, Frankfurt/Main, Germany

## Abstract

**Background:** In accordance with the German Infection Protection Act, the treatment and handling of laundry was checked by the Public Health Department in 2016 in all Frankfurt nursing homes with special focus on the staff’s clothing.

**Methods:** On-site visits and surveys were conducted in all 44 nursing homes in Frankfurt/Main, Germany, and random microbiological examinations of 58 reprocessed and 58 already worn protective gowns were performed to determine the numbers of the colony forming units (cfu) and microbiological differentiation of the pathogen species.

**Results:** 41 (93%) of the 44 homes tested had contracted a certified laundry service. 23 (52%) of the homes also ran a laundry of their own; in 21 of these, laundry was reprocessed and disinfected in an industrial washing machine. Regular technical or microbiological tests were carried out in 16 or 12 of the home-owned laundries, respectively. Only 31 homes (70%) provided uniforms for their employees. The staff’s clothing was processed in 25 homes by the external laundry, in 9 homes by the internal laundry, and in 12 homes, the nursing staff had to do this privately at their own home.

Used coats exhibited significantly higher contamination than freshly prepared ones (median: 80 vs. 2 cfu/25 cm^2^; P 95 percentile: 256 cfu vs. 81 cfu/25 cm^2^). Clothing prepared in private homes showed significantly higher contamination rates than those washed in the certified external laundry or in the nursing homes themselves (Median: 16 cfu/25 cm^2^ vs. 0.5–1 cfu/25 cm^2^).

**Conclusion:** Considering various publications on pathogen transfers and outbreaks due to contaminated laundry in medical facilities, the treatment of laundry, in particular the uniforms, must be given more attention, also in nursing homes for the elderly. The private reprocessing of occupational clothing by the employees at home must be rejected on hygienic principles, and is furthermore prohibited by law in Germany.

## Zusammenfassung

**Hintergrund:** Im Rahmen der im deutschen Infektionsschutzgesetz verankerten infektionshygienischen Überwachung wurden im Jahr 2016 in allen Frankfurter Alten- und Pflegeheimen die Aufbereitung und der Umgang mit der Wäsche mit besonderem Fokus auf der Dienstkleidung der Mitarbeiter überprüft.

**Methoden:** Mittels Vor-Ort-Begehung, Befragung in allen 44 Altenpflegeheimen und stichprobenartiger mikrobiologisch-krankenhaushygienischer Untersuchung von 58 frisch aufbereiteten und 58 getragenen Schutzkitteln mittels Kontaktkulturen, Nachweis der Koloniezahl und mikrobiologischer Erregerdifferenzierung wurde der Hygienestatus bewertet.

**Ergebnisse:** 41 (93%) der überprüften 44 Heime hatten eine zertifizierte Fremdwäscherei unter Vertrag. 23 (52%) der Heime verfügten auch über eine eigene Wäscherei, 21 davon bereiteten die Wäsche desinfizierend in einer Industriemaschine auf. Regelmäßige technische Prüfungen wurden in 16, regelmäßige bakteriologische Untersuchungen in 12 der heiminternen Wäschereien vorgenommen. Nur in 31 Häusern (70%) wurde die Dienstkleidung für das Pflegepersonal vom Haus gestellt. Die Dienstkleidung der Pflegemitarbeiter wurde in 25 Häusern in der externen und in 9 Häusern in der internen Wäscherei aufbereitet, 12 Häuser ließen die Dienstkleidung des Pflegepersonals von diesem privat zu Hause waschen.

Gebrauchte Kittel wiesen eine deutlich höhere Kontamination auf als frisch aufbereitete (Median: 80 vs. 2 KBE/25 cm^2^; P 95 percentile: 256 vs. 81 KBE/25 cm^2^). Die privat zu Hause aufbereitete Dienstkleidung wies deutliche höhere Kontaminationsraten auf als die in der Wäscherei aufbereitete (Median: 16 KBE/25 cm^2^ vs. 0,5–1 KBE/25 cm^2^).

**Schlussfolgerung:** Angesichts verschiedener Publikationen zu Erregerübertragungen und Ausbrüchen durch kontaminierte Wäsche in medizinischen Einrichtungen muss der Aufbereitung der Wäsche, insbesondere der Dienstkleidung, mehr Beachtung geschenkt werden, auch in Altenpflegeheimen. Die Aufbereitung von Dienstkleidung durch die Mitarbeiter privat zu Hause ist fachlich abzulehnen und juristisch untersagt.

## Introduction

Hand hygiene is the most important measure of infection prevention [1]. Basic hygiene also focusses on the appropriate reprocessing of medical products and the disinfection of surfaces in the patient’s vicinity [2], [3]. Many studies clearly demonstrate the importance of these measures. In comparison, the laundry – professional/protective clothing of the staff and laundry for patients/residents – has to date received less attention [4], [5].

In the context of the hygienic monitoring of nursing homes according to the Infection Protection Act [6], the handling of laundry and the reprocessing of the staff’s protective equipment were examined in 2016 by the Public Health Department, Frankfurt am Main, Germany. Nursing homes are increasingly deciding against having their laundry disinfected at external treatment facilities, and instead expect the employees of the nursing home to do it themselves at their own home. 

## Materials and methods

All 44 homes were visited by employees of the Public Health Department. Using a checklist (Table 1 (Tab. 1)), the structural quality of the laundry preparation – both residents’ and staff’s laundry – was tested. 

In addition, the homes were asked to participate in a voluntary examination of the contamination of nurses’ gowns and quality control of the preparation of the nursing staff’s gowns. This was tested in 12 homes: 4 homes each with external preparation of laundry, four homes with an internal laundry service, and in four homes, the employees washed their own gowns at home. In each of these nursing homes, a freshly prepared gown and a used gown from 5 staff members were tested, using contact plates and following microbiological analysis from the abdominal pocket area.

The contact plates (Caso-Agar with lecithin, tween 80 and histidine; Xebios, Dusseldorf, Germany) were processed at the Institute of Medical Microbiology and Hygiene of the University Hospital of Frankfurt, Germany, immediately after sampling. All laboratory testing was performed under strict quality-controlled criteria (laboratory accreditation according to ISO 15189:2007; certificate number D–ML–13102–01–00, valid through January 25th, 2021). Microbiological diagnostics was performed using standard microbiological techniques including matrix-assisted laser desorption ionization time of flight analysis (MALDI–TOF) and VITEK2 technology (bioMérieux, Nürtingen, Germany) [7], [8], [9].

Data were analyzed using SPSS, Version 15 and nonparametric Mann-Whitney and Kruskal-Wallis tests. 

## Results

### Survey and on-site inspection 

41 of the homes had a contract with an external laundry, all of which possessed a valid laundry certificate. 23 (52%) homes also run their own in-house laundry. In 21 of these 23 in-house laundries, clothing was washed using a disinfecting process in industrial washing machines. In 12 homes, this procedure was regularly microbiologically tested, and the washing machines were regularly maintained in 16 houses. 16 homes ensured that the sealing gasket was disinfected when the laundry was removed, in order to avoid recontamination of the treated laundry. Adequate black-and-white separation and the wearing of protective clothing were ensured in-two thirds of the homes. Only in two houses sorting of collected dirty laundry was not excluded.

In only 31 (70.5%) nursing homes occupational clothing for the nursing employees was provided by the employer. Five or ≥7 tunics per person were provided in 22.7% or 27.2%, respectively, of the homes. Regarding trousers, 5 per person were available in 34% of the homes. About one-third of the homes did not give full particulars on this question. 25 (56.8%) homes had their nurses’ working clothes reprocessed at the external laundry, in 7 (15.9%) homes they were reprocessed at the in-house laundry, and in 12 (27.3%) homes, the employees had to wash their occupational clothing in their own home (multiple answers possible).

Most of the nursing homes provided reprocessing of staff clothing at the external laundry under contract: 79.5% of the residents’ laundry, 79.5% of the kitchen staff’s clothing, 68.2% of the housekeeping staff’s clothing and 56.8% of the nursing staff’s. The in-house laundry prepared 25% residents’ linen, 13.6% kitchen staff’s clothing, 15.9% housekeeping staff’s and 15.9% nursing staff’s clothing. The nursing staff most often had to wash their professional garments a home (27.3%), housekeeping staff and kitchen staff less often (20.5% and 9.1%).

In 42 homes, the laundry was protected from recontamination (by the residents, for example) during distribution. In 40 homes, laundry storage was also protected, but only in 38 homes was the freshly delivered laundry stored correctly as well (Table 1 (Tab. 1)).

### Microbiological examination of nurses’ work tunics 

**Figure 1:**
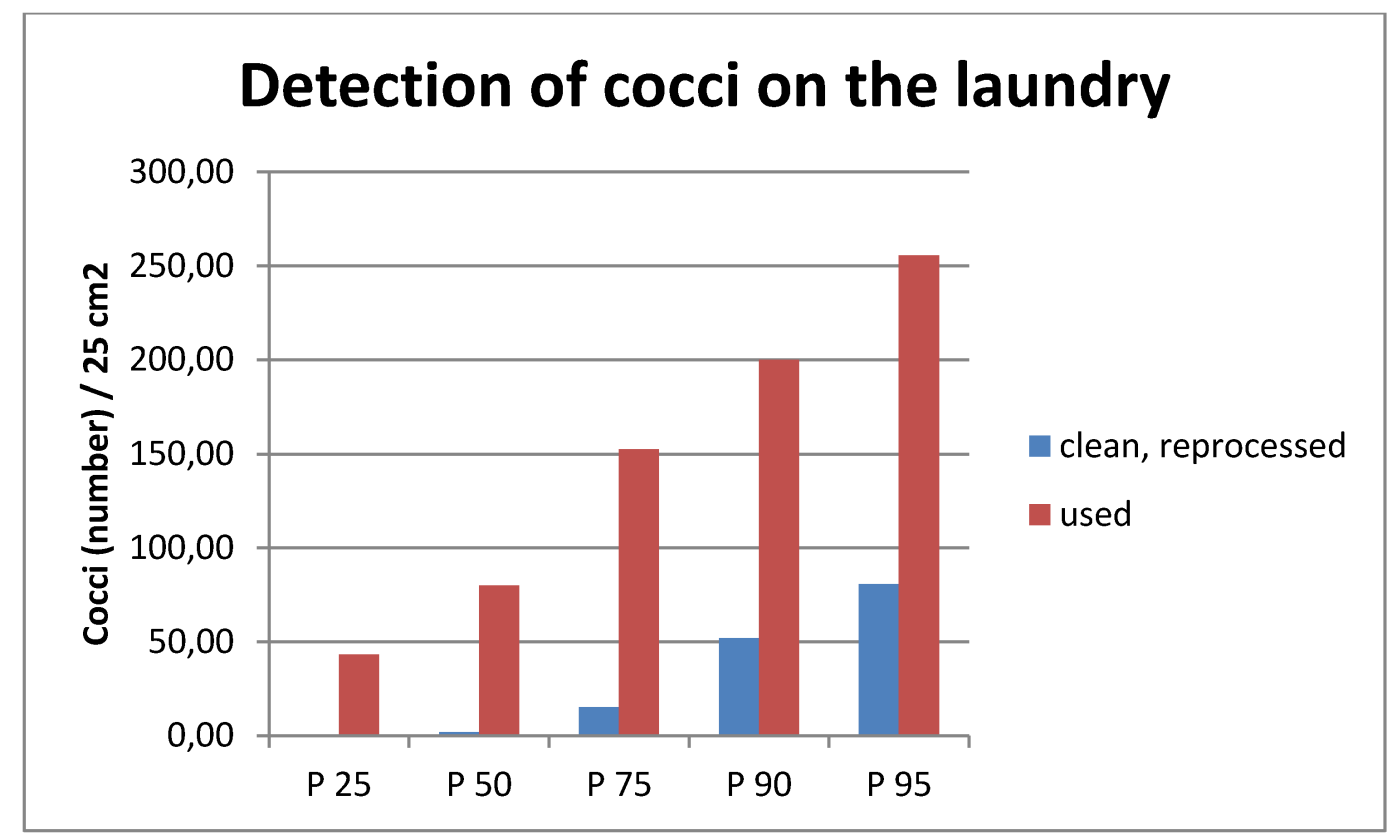
Microbiological examination results of clean and used gowns of nursing personnel (CFU/25 cm^2^)

A total of 116 samples were examined, 58 samples from used and 58 samples from washed coats. The contamination of the used gowns was, as expected, significantly higher than that of the freshly reprocessed ones (Mann-Whitney U-test p<0.000), with the median value of the used gowns about 40 times, the P 75 about 10 times, and the P 95 about 3 times higher than the respective percentile values of the clean linen (Table 2 (Tab. 2), Figure 1 (Fig. 1)).

Facultative pathogens were detected on 20 of the used/worn gowns and on 2 of the freshly prepared gowns; the latter were not protected in storage. *Staphylococcus aureus* and other staphylococci were the most frequently detected species, in addition to 6x *Acinetobacter baumannii*. In no case did the detected pathogens exhibit any specific resistance to antibiotics.

**Figure 2:**
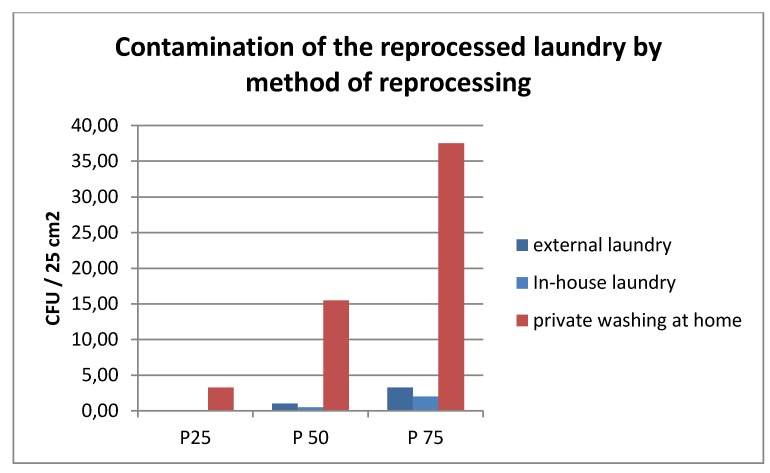
Microbiological examination results of the nursing staff’s prepared gowns by method of reprocessing – external laundry, in-house laundry, washing at home (percentile)

There were no differences in the level of contamination of the nurses’ clothes between those reprocessed by a disinfecting washing process in a certified laundry and the in-house laundry, whereas contamination levels of privately washed garments were higher (percentiles; Kruskal-Wallis test p<0.000). However, a very high maximum value of 200 cfu/25 cm^2^ was found on the externally processed gowns, thus yielding a very high mean value, which was in the same range as home-privately washed laundry (Table 3 (Tab. 3), Figure 2 (Fig. 2)).

Regarding individual results, it was found that 15 of 18 externally reprocessed gowns exhibited ≤10 cfu/25 cm^2^, but two of them had ≥150 cfu/25 cm^2^. In 19 of 20 samples of the in-house prepared laundry, ≤10 cfu/25 cm^2^ were detected, with a maximum value of 17 cfu/25 cm^2^. All of the privately prepared clothes were contaminated, 8 of them exhibiting ≤10 cfu/25 cm^2^ and 12 between 13 and 77 CFU/25 cm^2^.

When looking at the individual sample results from the different nursing homes, it is apparent that with external and internal treatment, the contamination levels were less than 20 cfu/25 cm^2^, with the exception of nursing home 23 (external laundry). In contrast, in most of the samples from the nursing homes where the staff laundered their clothes privately, a higher contamination was found, although one nursing home also performed well here (nursing home 37). 

## Discussion

§ 36 of the Infection Protection Act stipulates that, inter alia, nursing homes must set their standards for infection prevention in hygiene plans. The health authorities are obliged to supervise the nursing homes regarding their hygiene standards [6]. The Public Health Office of Frankfurt am Main has conducted standardized inspections of the nursing homes for many years and publishes the anonymous and summarized results. Whereas in the 1990s, many or all hygiene areas were controlled [10], [11], in the last few years, special areas have also been addressed, for example, disinfection of surfaces in 2011 [12] and the handling of urinary catheters in 2015 [13]. In 2016, the hygienic handling of laundry was checked. 

This survey was done considering publications on the contamination of the coats of doctors and nurses in the course of their activities and was based on the relevant recommendations on the proper handling of laundry of patients and staff [4], [5].

As early as 1991, contamination of doctor’s overalls – especially with *Staphylococcus aureus* – was reported [14]. Bacteria that were transferred from the patient or patient’s environment to the hands of the staff are often also detectable on the coats [15]. This was particularly evident in the care and treatment of patients with multi-drug resistant organisms (MDRO) [16], [17]. In a study conducted in Lusaka, Africa, 94 of 107 investigated white coats (72.8%) were microbially contaminated after a short time [18]. In a clinic in Tanzania, the rate of contamination of white coats was in the same range (132/180; 73.3%), with staphylococci being most frequently detected [19]. On more than 75% of the white coats of 100 medical students in India, S. aureus was found [20]. 

Very high contamination rates were also detected in gowns of nursing staff. In a US study, the cfu per square inch averaged 1,246 and 5,795 after a 12-hour day or night shift, respectively [21]. In another study, 94% of 300 doctors’ gowns were contaminated with pathogens, with higher levels of contamination found in the abdominal region than in the pocket and the sleeve [22]. This was recently confirmed in a German study, which exhibited even higher contamination on pockets than on the front (abdomen), in doctors’ as well as with nursing staff’s gowns [23]. In some investigations, MDRO were also reported on used gowns: in Maryland, USA, 23% of the 149 medical gowns tested were contaminated with *S. aureus* and 18% of them with MRSA [24]. In one hospital in Israel, where the employees claimed to observe good hygiene and change their gowns every day, 6% of the doctors’ white coats and 14% of the nurses’ coats were contaminated with MDRO [25]. Even after just one short care-visit of patients with MRSA or vancomycin-resistant enterococci (VRE), clothing was contaminated with MRSA (4.3% 4/94) or with VRE (6.2%, 6/94) [17]. In another study, *Clostridium difficile* was found as well [26]. In 23% of 149 medical students’ used gowns tested, *Staphyloccocus aureus* was detected, of which 18% were MRSA [27]. MRSA have been found on clothing, especially in the abdomen and pocket area [28]. Such contamination can lead to pathogen transfer and infection outbreaks [29].

In one hospital with an outbreak of mucor mycosis with severe fungal infections of the skin, including deaths, the contamination of bed linen with *Rhizopus* spp. was identified as the cause. The laundry, which had been properly prepared, had been transported unsealed and was apparently polluted via air contamination due to construction work in the vicinity of the laundry [30]. Care must therefore be taken to ensure correct, recontamination-protected transport and storage. 

There are some indications that the microbial load on polyester-cotton blend gowns are 60% higher than that on the polyester gowns [31]. In terms of newly developed fabrics, a recent pilot study compared the contamination rates of silver-hybrid clothing with that of standard textile clothing. In samples taken from jackets and pants of 10 emergency workers on day 0 (pre-service), day 3 and day 7 after use, no significant difference in the extent of microbial contamination was detected between these two materials. The authors concluded that a larger sample size is required to verify this result [32].

For the reprocessing of patients’ (residents) and staff’s laundry, recommendations have been published by the Commission on Hospital Hygiene and Infection Protection (KRINKO) [33], the German Society for Hospital Hygiene [4] and the Professional Association for the Health System [5]. The first KRINKO recommendation , “Hygiene requirements for hospital laundry, hospital laundering and washing and the requirements for contracting hospital laundry out to commercial laundries”, was published in 1979 [33]. The Professional Association for the Health System [5] distinguishes between professional and protective clothing: “Professional clothing is worn instead of or in addition to the private clothing at work. It may be worn as status symbol or uniform without a special protective function for the wearer. 

Protective clothing is any clothing designed to protect employees from harmful effects at work or to prevent contamination of personal clothing by biological agents” [5]. According to [5], protective clothing must be disinfected by the employer as well as contaminated working clothes. The German Society for Hospital Hygiene (GSHH) also states: “Probably contaminated clothing is to be treated by the employer in the right way, i.e. using effective disinfecting procedures.” [4].

As early as 2011, data on the handling of laundry in 22 nursing homes in North Rhine-Westphalia were published. There, too, only 14 (64%) of the homes provided the staff’s clothing and had them washed by the employer; in 4 nursing homes (18%), the nursing staff had to wash their gowns at their own home [34]. In our study, the proportion of homes in which nursing staff had to wash their gowns at their own home was 27% and thus even higher. However, the nursing homes in North Rhine-Westphalia had voluntarily participated; therefore a bias cannot be ruled out, while our data from Frankfurt encompassed all the nursing homes in the city (100% response).

 Because our surveys show that nursing homes less and less often send their laundry to an external service for disinfection, and expect their employees to do this at their own home, we carried out microbiological tests of used and freshly washed coats. The aim was to determine whether the cleanliness of the laundry differed when different processing methods were used (external disinfecting treatment by a certified laundry, in-house disinfecting reprocessing, and private washing at home).

The present investigation is a pilot study with a small number of samples, which nevertheless produced remarkable results. As expected, higher contamination was detected on worn clothing than on freshly washed clothes. This shows that the chosen method is generally suitable to answer the study question. To the authors’ knowledge, unfortunately only studies with coats of medical and nursing staff in hospitals (all studies above) are available in the literature, and none from nursing homes. Because of different methods, the comparability of the data is limited. We examined the used gowns at shift changes; the gowns were usually worn for two shifts. In other studies, the gown was tested after up to several days of wearing. In accordance with other studies, cocci and staphylococci were frequently found, but MDRO were not found in the present study. This is astonishing, as we know of a high prevalence of MRSA (6–9%) and multi-drugresistant Gram-negative bacteria (12–19%) among residents of nursing homes in the Rhine-Main region [35], [36]. This result could be caused by the small number of examined used gowns, or the fact that additional protective gowns were worn during contamination-intensive care with the residents. 

*Staphylococcus aureus* and *Acinetobacter baumannii* were detected on the clothing of the nursing staff. In principle, this allows the conclusion that pathogens, which are classified by the WHO as highest priority [37], can be found on working gowns of the staff and can thus become the starting point of transmissions.

Gowns washed privately at home exhibited significantly higher contamination levels than those reprocessed in the nursing homes or in external laundries. This is in accordance with other publications on the reprocessing of textiles in private households [38] and laundry treatment in commercial laundries [39]. However, the highest individual loads were found in a coat from a home with an external laundry. This could be caused by contamination due to improper transport or storage. Two conclusions can be drawn from this data: first, the nursing staff’s clothing must be properly washed and disinfected, either by certified companies or via a disinfecting treatment in the institutions themselves (industrial washing machines, controlled disinfecting processes). Second, recontamination of the freshly prepared laundry must be avoided by safe, protected storage and distribution.

## Notes

### Competing interests

The authors declare that they have no competing interests.

## Figures and Tables

**Table 1:**
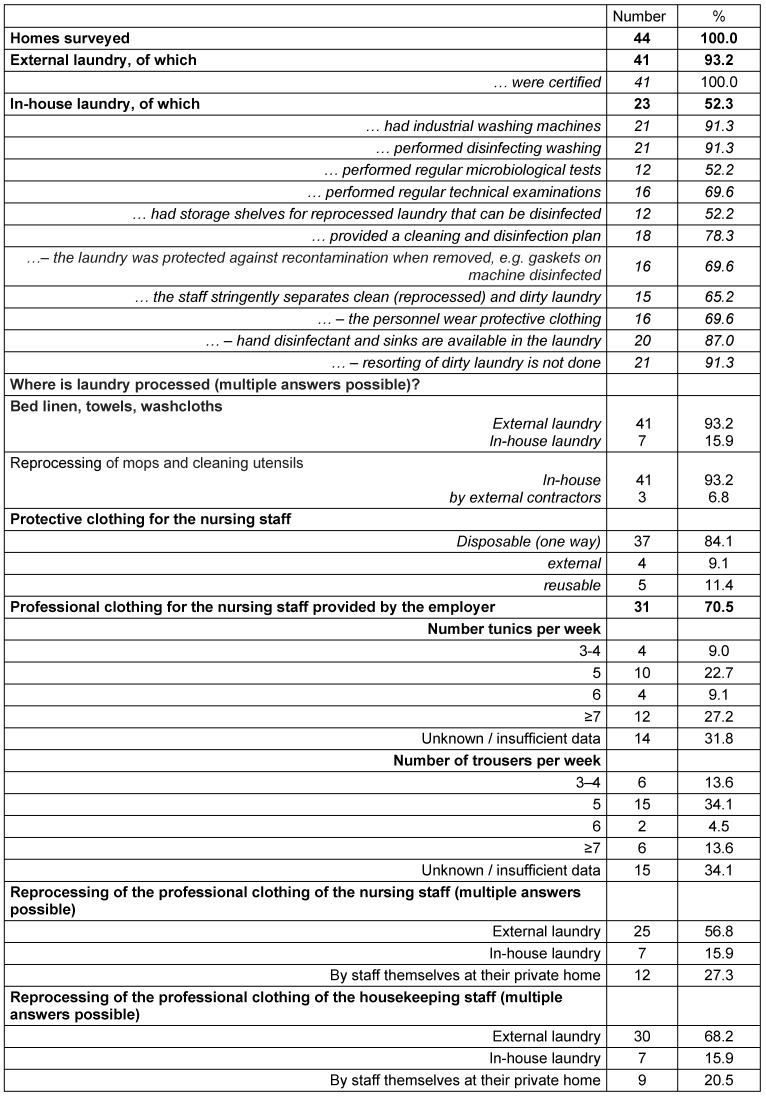
Data on the laundry processes used by the 44 nursing homes in Frankfurt am Main, Germany

**Table 2:**
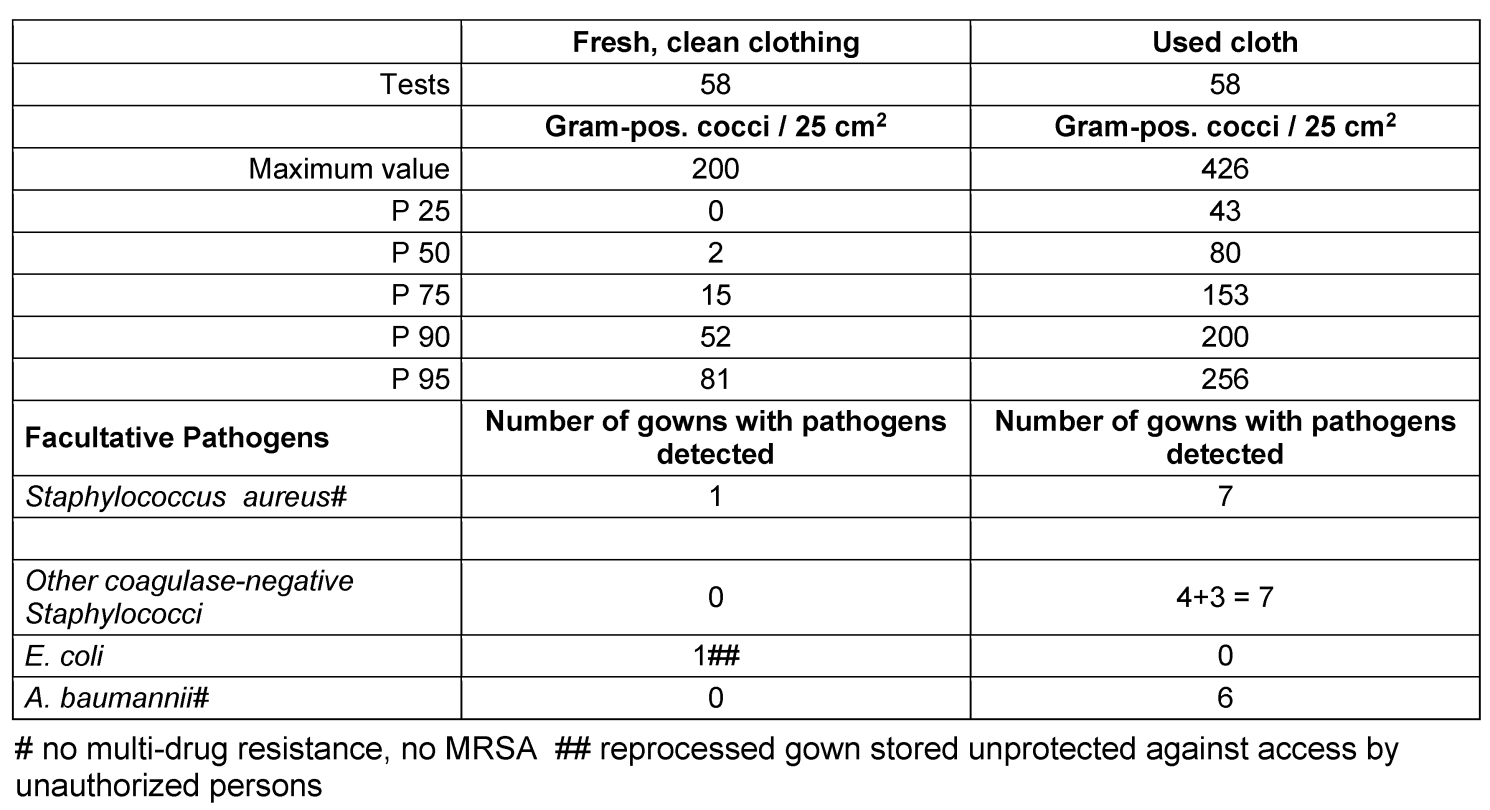
Results of the microbiological examinations of clean and used nursing gowns

**Table 3:**
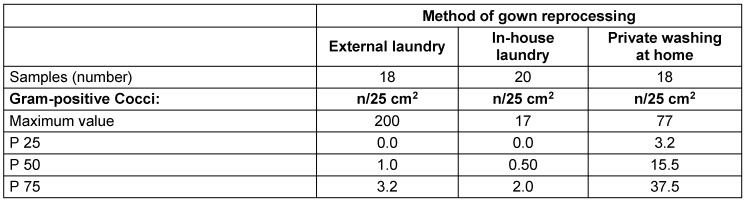
Microbiological examination results of the nursing staff’s prepared gowns by method of reprocessing – external laundry, in-house laundry, washing at home
